# New York Odontological Society

**Published:** 1892-05

**Authors:** 

**Affiliations:** New York Odontological Society


### Reports of Society Meetings.

### NEW YORK ODONTOLOGICAL SOCIETY.

A REGULAR meeting of the New York Odontological Society was held Tuesday evening, February 16, 1892, at the New York Academy of Medicine, No. 17 West Forty-third Street, with the President, Dr. Woodward, in the chair.

The minutes of the previous meeting were read and duly approved.

### INCIDENTS OF OFFICE PRACTICE.

*Dr. Howe.*—I have discovered from conversation with several of my friends that they have not read a paper published in the October number of the INTERNATIONAL DENTAL JOURNAL, by Dr. E. A. Stebbins, on the treatment of caries of the teeth by nitrate of silver. I rise to call the attention of those present to that paper, and urge upon all who hear me to read it and begin to put the suggestions into practice. First, that they may prove the method, and secondly, that they may get the experience to discuss the subject at a later date. I have very good hope that Dr. Stebbins will, within the year, come to New York, and perhaps show us some patients whose teeth he has treated with nitrate of silver. The paper, and his series of experiments, have impressed me as being very valuable, and containing many very important suggestions in our work.

*Dr. Littig.*—Would it not be well for Dr. Howe to suggest how that is used?

*Dr. Howe.*—It seems unnecessary that I should take up the time of the meeting in explaining what is contained in the paper. I urge the reading of the article; it will be more satisfactory than any report I could give of its contents.

*Dr. Dwinelle.*—I have a little matter that I would like to lay before you. A few weeks ago I was making a very important filling,—one of those cervical fillings that extend under the gum,—and when about half through the operation, it got wet. I disliked to give it up, as it was a very important one, and I was tempted to go forward. I was using the new mat gold, brought out by the Johnsons. Though completely submerged in water, I renewed the operation by adding gold to that already consolidated. I directed my assistant, after annealing, to lay one flat piece after another upon it, and then I used a little engine-burnisher in the shape of a tomato, which I call my hammer-burnisher. I applied it to the gold, commenced rotating it, and, to my surprise, I found it adhered. This rotating principle was so effective that it induced adhesion. I proceeded, and completed the operation very successfully. I showed it to some of my friends, and they pronounced it solid and thoroughly integrated. I have some specimens here that I will show you. They have been tested by instruments accompanied with a great deal of pressure. I do not recommend filling teeth under water, but when you get caught, as I did here, I think it well to take advantage of the fact that you can, by using a burnisher of that character, force the gold into itself. You can integrate it, so to speak, and make a success out of a failure. I do not recommend filling teeth under water, as I said before, but it is very desirable, under circumstances.such as these. I have made sections of these water specimens, and find they endure the most practical test.

There is another subject I want to talk about. In regulating teeth we often use wires. We connect the teeth with a twist, and that twist is forever untwisting. I have adopted an expedient that makes it very easy to do a difficult thing. I make my wires loop-wires. I connect them by a portion of solder, and in that way have much greater efficiency than under ordinary circumstances. I take two loops and bring them together, and have a mere speck of solder to connect them. Each time you commence "*de novo;*" you are solid from the foundation. I will have them passed around, and they will probably recommend themselves, and suggest the advantages to be derived from them over an ordinary twist. You will be surprised to learn how, by thus connecting the loops, it facilitates and gives permanency to the operation of wiring the teeth.

I saw a very beautiful burnisher in Dr. Northrop's case,—a tomato-burnisher, made of agate, I think,—which he obtained in Europe. A rotary instrument of that kind is effective in a great many ways. My experiments were very successful. They are as hard as any fillings that I have ever made.

I use copper wire, for I find it tougher than any other metal, It does not oxidize in the mouth, has no taste, and is always bright and clean.

*Dr. Bogue.*—Five years ago I put in a number of oxyphosphate fillings, Poulson's cement. I again saw the patient to-day, and I find those fillings still in the teeth. Adjoining the fillings are cavities larger than the fillings by a good deal, yet the fillings remain. They are a little roughened on the surface, but they are there, and protect the portion of the cavities not decayed. I speak of this because one or two gentlemen here have inquired what the cement was that I used. It is Paulson's, and is made in Hamburg, Germany; it ought to be easily procurable here.

Another thing that I have lately run across in one of the journals (I have forgotten who the author is), although it is an old remedy, is granulated chloride of zinc, for obtunding sensibility. It is so potent a remedy that I should like to have other gentlemen try it as well as myself. I find that where the patient is extremely timid, if I wish to get rid of sensibility, and have not the fear of destruction of the pulp, a little pure cocaine, or rather cotton dipped into carbolic acid, and then into powdered cocaine, will obtund the sensibility enough to put in granulated chloride of zinc with little or no pain. It deliquesces very speedily. In a minute and a half the insensibility of the cavity is often complete.

*Dr. Howe.*—Only in the pulp, did you say, Dr. Bogue?

*Dr. Bogue.*—I did not say; that is what I want you to share the responsibility of. I am not aware of ever having done harm to the pulp, but I cannot assert that it will not do harm. The granulated chloride of zinc is quite fine. It will stay in that condition just a little while. You keep it in an hermetically-sealed bottle. You can put a grain or so into smaller bottles at a time, but it will deliquesce and get into solid masses very quickly.

*Dr. Dwinelle.*—In a paper I wrote some time ago I recommended chloride of zinc, which has been suggested. I not only recommended using the salt pure on sensitive dentine, but enforcing its effectiveness by heat. I had steel bulbs of different kinds, which, after heating, were put into the cavity and literally cooked the inside of the tooth. This can be done with impunity. I never lost a nerve by the use of it.

*Dr. Ottolengui.*—I believe most of the gentlemen know that I have paid some attention to this subject. When I hear a statement made that anything put into a cavity will obtund sensitive dentine, I feel inclined to think that it will only do so sometimes. I had a tooth in my own mouth which needed filling, and as it was very sensitive, I prepared to endure the pain. My dentist said he could guarantee that my tooth would not hurt me at all if I were willing to submit to what he would recommend. He gave me about six drops of chloroform on a handkerchief, and asked me to inhale it until I felt that it was having an effect,—not to go to sleep with it, but to be able to tell him myself to go on. I did that, and I was very much surprised to find that the first effect is the same as that of nitrous oxide. I gave the doctor the signal, and was as conscious as I am now, and told him when the tooth became painful again; but by that time he had almost completed the work.

The point raised in my mind was, that it is claimed by surgeons that to operate under partial anæsthesia is more dangerous than when the patient is put thoroughly to sleep. We know chloroform is not safe to use. The question arises, however, whether the operation of cutting a tooth under so very powerful an anæsthetic is dangerous. I have used it on three patients in my own office, and it has been successful. That statement is believable, because we know that chloroform will obtund any pain, and it seems to me that if it is a safe operation, we have the solution of the problem in a nutshell. I thought it of sufficient importance to bring up before this meeting, and I would like some one to tell me whether there is any risk about it. I know that so careful a man as Dr. Brockway has done it for years. Dr. Van Woert did it for me. I had heard of it, but did not believe it. I would be much indebted to any one who will tell me if it is dangerous. If it is not, we should all practise it. So little is necessary, and the control of pain is so admirable, that it would seem advisable to use it.

*Dr. Northrop.*—Does the speaker know anything of the history of Mr. Harper's daughter-in-law, who died under such circumstances?

*Dr. Ottolengui.*—Was the narcosis complete?

*Dr. Northrop.*—No; she merely took a small quantity of it to have a filling removed. She said her former dentist had been in the habit of giving her a little, and she insisted upon the dentist giving her a small quantity. She died of the effects.

*Dr. Ottolengui.*—I am very glad now that I know of this. I thought there was a danger, but I wanted some corroboration of it. I should think a single death of that kind would deter us from using it.

*Dr. Mills.*—The use of chloroform has been common to me since 1874. I use it as taught me by the late Dr. Riggs,—by alternate inhalations from a bottle. I only secure what is called the analgesic effect,—loss of sensation, without loss of consciousness. It has become so pain-saving in hundreds of cases, that I deem it a great blessing. I have yet to see a single case in which *any* unpleasant conditions have arisen. I have used it, personally, carrying the effect to loss of sensation, and directing the cutting of a very painful abscess without hurt. The operation was performed by the late Dr. Atkinson, who pronounced it a decided success. I published a paper, five years ago, in the *Archives*, giving in detail my practice, etc., with it.

*Dr. Weld.*—I wish to say that there is in one of the medical journals of this week an editorial on the subject regarding the use of chloroform and ether. We all know the effects are very different. The action of chloroform depresses the heart. The action of ether depresses the muscles of respiration. At the same time the man who wrote the paper—I forget his name—stated that in England the mortality as between ether and chloroform was about equal. It may be possible that a person may inhale a little chloroform and die from the depressing effect it has on the heart's action. At the same time, according to the record and according to the statistics, ether, which is supposed to stimulate the heart's action, so to speak, causes as many deaths as chloroform. I do not believe, unless there is some other reason for it, that chloroform does produce death very much oftener than ether. Perhaps some one can produce different statistics; but from what I have read, I feel very doubtful that the inhalation of chloroform will produce death *per se*.

*Dr. Dwinelle.*—It is well known that in former years it was used, under all circumstances, for the simplest operations; but latterly the views have changed in reference to it, and now it is never used unless necessary. I have given it in almost every known surgical operation. I was the first to do so in my vicinity in the country many years ago, but I rarely use it now. There seems to be no system about its use. Not long since I administered it to a physician's wife; he had used it previously, and the patient died on his hands; and yet we felt justified under the circumstances in giving it.

*The President.*—The subject before you is one of such importance that your executive committee has thought it wise to devote an evening to it. I will say that the Dental Protective Association has been very happy in the choice of one whom we are all pleased to welcome. No one will present this matter in a better light than Dr. Louis Jack, of Philadelphia.

(For Dr. Jack's paper, see page 321.)

DISCUSSION.

*Dr. Francis.*—When we see one like Dr. Jack, who has a very large office practice to look after, giving so much of his time in behalf of the profession, and are also aware of the fact that Dr. Crouse has given so much to the same object, it seems to me that the dentists of New York and elsewhere ought to become more interested in the Protective Union. Dr. Jack's reference to the old vulcanite suits brings forcibly to mind the time when I was one of the committee appointed to collect funds to defend dentists against the extortions of the Goodyear Company. I, however, found it very difficult to collect money sufficient to pay our expenses. I now urge every one in this room who is not a member of this Association to become one, and also to influence his professional friends who are not members to join us. Do not let the burden of the work fall upon the shoulders of two or three; but all do your share.

*Dr. Hill.*—In order to give this a practical turn, I suggest that every one in this room who is not a member of this Association give his name to the president or to the secretary. Let the secretary forward that name to Dr. Crouse, and then Dr. Crouse will send on the by-laws for him to sign, and he can send his ten dollars and become a member. New York is far behind in the matter of membership in the Association, and it is very unfortunate, because there is more money in this city than elsewhere. As we all know, Dr. Crouse and the others regret that New York and Connecticut do not take more interest in this matter. The money that is spent will be mainly spent here; and gentlemen should become members.

*Dr. Hoyt.*—I would suggest that any person who presents a name to the secretary should give ten dollars; that would be the safest way.

*Dr. Weld.*—Four or five months ago I knew nothing about the Association. Dr. Crouse sent on by-laws, and also a blank note. I do not know whether any other members of the profession received anything of that kind. It was about May or June. He said, "Fill out this note for three months, for ten dollars." When Dr. Crouse was here, I paid him the ten dollars. I presume there are other gentlemen here, perhaps young men, who would like to do the same thing. I think Dr. Crouse will accept a note for three months, as well as the ten dollars in cash.

*Dr. Howe.*—I am glad Dr. Weld spoke of the liberal manner in which the Dental Protective Association is willing to deal with members of the profession, in order that they may all have the opportunity of joining the Association, whether it is convenient to pay the ten dollars at the time or not. The Association offers to receive a note for the amount, and make the person a member of the society on his promise to pay. The benefits of the past work of the Association must be apparent to every one who knows anything about the relation of the Tooth-Crown Company to dentists, and the sentiment of common interest which is manifested by those who have worked for this end is shown in the fact that the probability is that the men who have done most for the Dental Protective Association have done almost nothing in the way of crown- and bridge-work. The number of members of the Association, although large, is not half or quarter what it should be. I do not know how many members New York has, but it certainly ought to be a great deal larger than it is. The young men of this city ought all to be members. When they realize that Dr. Crouse and others, like Dr. Jack, are working for this organization in an entirely disinterested way, they should show their appreciation more than they have done. The whole work has been accomplished in response to a desire to make dentists more united, giving them more of a common interest, and a common and united power.

*Dr. Ottolengui.*—There is not any subject in which I am more interested than this one. What I am going to say now is not intended boastfully, but I wish to tell my own experience with this Association, simply to show how much more can be done by working than by recommending and doing nothing yourself. When I joined the Dental Protective Association, I told Dr. Crouse that, having been convinced that his Association was doing good work, and having decided to join it, I would guarantee to get for him twenty-five members. He gave me twenty-five blanks, and I sent him fifteen members within two months. Since then I have sent him some more of the twenty-five; and when I hear that there are three thousand or four thousand in the Association, I am gratified. I want to point out some of the obstacles which I have met with in doing that work. While the charge made that New York is behind in membership is true, there is an explanation in it in this: That in proportion to the number of dentists in the State, we are greatly behind in the matter of membership in dental societies. Now, when a mass-meeting for the Dental Protective Association is arranged, as was done hurriedly last month, and when the hall is canvassed, the difficulty of getting new members lies in the fact that almost all the men are members already. Out of an attendance of between one hundred and fifty and two hundred, over one hundred and fifty declared that they had been enrolled. That is not a good field to work in; consequently the same thing, I prophesy, will occur again unless something is done in that direction.

It seems to me that some sort of effort should be made to get members for the Dental Protective Association from men who are not members of dental societies. I should be very glad if every one in this room would not wait until the mass-meeting, but would go there and say, "I have, during the last month, been able to persuade four or five to join the Association, and here is the money for them."

*Dr. Hill.*—I do not blame Dr. Crouse for being tired and disgusted with urging men to join this Association. In all the litigation that has been going on for years and years, a few men have paid the bills. At the last meeting the question was quite seriously discussed of not letting any more become members, on account of the absurdity of going around and having to coax men to join.

*Dr. Edward C. Kirk.*—I think anything I could say before this intelligent body of practitioners would be in the nature of carrying coals to Newcastle. Every dentist has seen the usefulness of this Association, and should embrace the opportunity of becoming a member. The question seems to me to be a strictly business one. It is a question whether you will fight your own battle and pay a big price, or whether you will pay ten dollars to the Association and let it fight for you. It is somewhat like the matter of life insurance. It is not necessary to question the legitimacy and correctness of life insurance from a business stand-point in the latter part of this nineteenth century. There is a point which Dr. Jack touched upon which seems important to me. No doubt it has been said many times before, but it will not hurt to emphasize it again; that is, the limitations of the Dental Protective Association. It has nothing whatever to do with the question of ethics. It is a question of deciding between patent-rights and wrongs. The Dental Protective Association concerns itself with patent-wrongs entirely, not whether dentists should hold patents or not, but merely with the validity of them. We are loaded down with innumerable patents which we believe have no legal foundation, and until such patents have had a court decision as to their validity, the owners of them cannot expect tribute from any who use them. It is this burden of unjust taxation which the dental profession has suffered and which the Association proposes to remove.

There is one other point that may have restrained a certain body of men from joining the Association. I have reason to believe, from such observations as I have made in reading and in the discussions which I have listened to, that on the ethical question as to whether it is right or wrong for a dentist to hold a patent, the profession is not only divided, but there is a large class of men who in their hearts, if not openly, are in favor of patent-rights in dentistry, and I believe the impression has gotten abroad that the Association exists simply to carry on a crusade against patentees. This, I believe, is not so; but, whether it does or whether it does not, it is morally certain that it cannot get back of the organic law of the land which has created the patent system. If a man has a legal right to his patent, if it is valid, he can operate under it. We may create a moral sentiment against him, but, legally, we cannot take it away from him. In any case of litigation on the part of the Association involving the validity of a patent, if the patentee wins this suit, his patent is, by reason of the decree in his favor, just that much stronger.

*Dr. Dwinelle.*—The effectiveness of combination to resist a common enemy was very well illustrated in the old Dental Protective Union, which was in vogue a number of years ago. I had the honor of being its first president, and believe I am president of it now. We were a very formidable enemy to our opponents, and Bacon once told me, after the matter had passed, how very near we came on one occasion to defeating their entire efforts. If we had known the vantage-ground we occupied, we would have been successful. As has been suggested here to-night, the old motto, that "in union there is strength," has never been verified more completely than in this "Protective Association." It is a very complete exemplification of it. We were not as well organized then as we have been since, and the consequence was that the burden fell upon a few. I can testify to that; and even could show the figures. Out of my own private purse I expended about nine thousand dollars. Other dentists spent a great deal. It is put on a wiser basis now, to have the cause more common, more extended in its reach. The moral influence, as well as the pecuniary, is better. Crouse is right, and he ought to be sustained to the utmost. I hope we shall surprise him by the liberal share we shall take in the matter. I think there is a great deal of wisdom manifested in the management of the organization. We profit by experience, and I believe that we shall prove so formidable in our efforts that we shall utterly annihilate our common enemy, so that we never need fear him or his unjust and unholy cause in the future.

Every individual in the profession is under the greatest obligation to Dr. Crouse for the sacrifices he has made for us, and for the great persistency and ability he has displayed, not only in defence of our cause, but in the formidable *aggression* which he has seen fit at times to mete out to our common foe.

I speak feelingly on this subject, for, as I have intimated, I had considerable experience in a former "Dental Protective Union." Had it been carried on and been under the wisdom and direction of Dr. Crouse, instead of the two lawyers we employed, we would never have been obliged to discharge the one for his dishonesty, and the other for threatening to go over to the enemy, unless we gave him an additional fee of ten thousand dollars. This latter individual, although the dentists had paid him five thousand dollars already, held Drs. Walter Roberts, J. B. Newbrough, and myself personally responsible, and sued us for ten thousand dollars and costs. We finally beat him, after proving by three witnesses the infamous threat he had made.

I mention this to show the kind of vicissitudes we passed through in those days, and which Dr. Crouse, in his wisdom, is continually protecting us from, with a very small outlay of time and money.

I trust the time is not far distant when we as a profession will give a substantial recognition of our appreciation of our obligations to Dr. Crouse.

A vote of thanks was moved to Dr. Jack, and carried unanimously.

Adjourned to banquet hall.

John I. Hart, D.D.S.,
*Editor New York Odontological Society.*

